# Mortality attributable to *Plasmodium vivax* malaria: a clinical audit from Papua, Indonesia

**DOI:** 10.1186/s12916-014-0217-z

**Published:** 2014-11-18

**Authors:** Nicholas M Douglas, Gysje J Pontororing, Daniel A Lampah, Tsin W Yeo, Enny Kenangalem, Jeanne Rini Poespoprodjo, Anna P Ralph, Michael J Bangs, Paulus Sugiarto, Nicholas M Anstey, Ric N Price

**Affiliations:** Global Health Division, Menzies School of Health Research and Charles Darwin University, PO Box 41096, Casuarina Darwin, NT 0811 Australia; Centre for Tropical Medicine, Nuffield Department of Medicine, University of Oxford, Oxford, UK; Papuan Health and Community Development Foundation, Timika, Papua, Indonesia; District Health Authority, Timika, Papua, Indonesia; Department of Paediatrics, Faculty of Medicine, Gadjah Mada University, Yogyakarta, Indonesia; Division of Medicine, Royal Darwin Hospital, Darwin, Australia; Public Health & Malaria Control Department, International SOS, PT Freeport Indonesia, Kuala Kencana, Papua, Indonesia; Department of Entomology, Faculty of Agriculture, Kasetsart University, Bangkok, 10900 Thailand; Mitra Masyarakat Hospital, Timika, Papua, Indonesia

**Keywords:** Plasmodium, vivax, Malaria, Mortality, Indonesia, Papua

## Abstract

**Background:**

*Plasmodium vivax* causes almost half of all malaria cases in Asia and is recognised as a significant cause of morbidity. In recent years it has been associated with severe and fatal disease. The extent to which *P. vivax* contributes to death is not known.

**Methods:**

To define the epidemiology of mortality attributable to vivax malaria in southern Papua, Indonesia, a retrospective clinical records-based audit was conducted of all deaths in patients with vivax malaria at a tertiary referral hospital.

**Results:**

Between January 2004 and September 2009, hospital surveillance identified 3,495 inpatients with *P. vivax* monoinfection and 65 (1.9%) patients who subsequently died. Charts for 54 of these 65 patients could be reviewed, 40 (74%) of whom had pure *P. vivax* infections on cross-checking. Using pre-defined conservative criteria, vivax malaria was the primary cause of death in 6 cases, a major contributor in 17 cases and a minor contributor in a further 13 cases. Extreme anaemia was the most common primary cause of death. Malnutrition, sepsis with respiratory and gastrointestinal manifestations, and chronic diseases were the commonest attributed causes of death for patients in the latter two categories. There were an estimated 293,763 cases of pure *P. vivax* infection in the community during the study period giving an overall minimum case fatality of 0.12 per 1,000 infections. The corresponding case fatality in hospitalised patients was 10.3 per 1,000 infections.

**Conclusions:**

Although uncommonly directly fatal, vivax malaria is an important indirect cause of death in southern Papua in patients with malnutrition, sepsis syndrome and chronic diseases, including HIV infection.

## Background

Almost three billion people are estimated to be at risk of *Plasmodium vivax* malaria, the vast majority of whom live in south and southeast Asian countries [1,2]. In endemic regions, this relapsing disease is responsible for substantial morbidity, mostly associated with recurrent bouts of fever, anaemia [3] and adverse pregnancy outcomes [4,5]. *Plasmodium vivax* is not generally regarded as sufficiently virulent to cause death. Notwithstanding this benign reputation, over the last two to three decades, there have been multiple case studies of fatal vivax malaria [6,7]. More recently, hospital and outpatient surveillance systems have shown that *P. vivax*-associated mortality may be occurring with greater frequency than previously thought or reported [8-11].

In Papua, Indonesia, *P. vivax* infections exact a considerable toll [8,10]. This reflects the high risk of multiple recurrences, the presence of high-grade chloroquine-resistance [12], comparatively high-level endemicity [13] and a large proportion of relatively non-immune migrants in the area [14]. The fatality of hospitalised patients with *P. vivax* parasitaemia in Timika, southern Papua, has been estimated to be similar to that of patients with *P. falciparum* infections (1.6% versus 2.2% respectively) [10]. In the present study we aimed to determine the extent to which *P. vivax* contributed to those deaths by conducting an audit of the clinical records of all patients with pure *P. vivax* infections who died at Mitra Masyarakat Hospital between January 2004 and September 2009.

## Methods

### Study site

The geography, climate and demographics of Mimika District and its capital city, Timika, have been described elsewhere [4,10]. Our study was based at the Mitra Masyarakat Hospital (Rumah Sakit Mitra Masyarakat, RSMM), which, until November 2008, was the only referral hospital in the south of Papua. This facility provides treatment to all patients – free of charge for indigenous Papuan residents and at a nominal cost for non-Papuans. The hospital has 101 beds, a 24-hour emergency department, an active outpatient ‘polyclinic’, and a high care unit with facilities for intravenous infusions but not mechanical ventilation. Radiological services include X-ray and ultrasonography. Laboratory facilities are also available for haematologic and biochemical analysis and basic microscopy, but not for microbiological culture. In addition to the hospital, there is also a network of primary health clinics distributed throughout the community. An established community surveillance network covers 14 of these clinics and collects data on the total number of blood smear examinations and the number of malaria positive films read on a weekly basis.

Malaria transmission in Mimika District is generally restricted to lowland areas below 1,500 m elevation where it is associated with efficient mosquito vectors, primarily the *Punctulatus* group. The estimated average annual incidence of clinical and asymptomatic malaria is 876 episodes per 1,000 people, 46% due to *P. falciparum* and 39% due to *P. vivax* [13]. The point prevalence of asexual parasitaemia in the community has been estimated at 16.3% (7.7% for *P. falciparum* and 6.4% for *P. vivax*) [13]. Prior to March 2006, hospital guidelines recommended oral quinine plus a 14-day regimen of primaquine for the treatment of patients with uncomplicated vivax malaria. After 2006, quinine was switched to dihydroartemisinin-piperaquine (DHP) for all malaria species. Severe malaria was treated with intravenous quinine prior to May 2005 and intravenous artesunate thereafter.

### Prospective hospital surveillance procedures

The methods of malariometric surveillance at RSMM hospital have been described elsewhere [10]. Hospital guidelines dictate that all inpatients should have blood taken for malaria microscopy at the hospital laboratory. In a minority of cases, blood smears are also read by local expert research-based microscopists. Slides are declared negative after review of a minimum of 100 high-power microscope fields and if parasitaemia is present the density is scored semiquantitatively as 1+ to 4+. In 2004, a random sample of 1,083 positive slides from the hospital laboratory was re-examined by an independent expert microscopist with more than 10 years of experience. The overall concordance between initial and subsequent readings was 90% (979/1083). In 1.7% (18/1,083) of the slides, the second reading was negative and in 4% (38/922) of cases, slides reported as monoinfections were, in fact, found to be mixed-species infections. In a minority of cases blood was tested for histidine-rich protein 2 (HRP2) using the Paracheck Pf® (Orchid Biochemical Systems, Goa, India) rapid diagnostic test (RDT).

With the aid of an automated alert system, a research nurse searches the wards for any patient with parasitaemia at least once per day. A research physician then reviews patients to determine the presence of severe disease according to the World Health Organization (WHO) criteria [10,15]. Respiratory distress (oxygen saturation (off supplemental oxygen) of less than 94% or an age-adjusted elevation in respiratory rate (>32 breaths per minute in adults, >40 breaths per minute in children 5- to 14-years old, >50 breaths per minute in children 2-months to 5-years old, and >60 in babies less than 2-months old), coma (Glasgow Coma Score <11 or Blantyre Coma Score <3) and anaemia (haemoglobin <5 g/dL) are routinely assessed, whereas assessment of the other severity criteria is less systematic as they generally rely on more specialised tests or examinations that are only conducted if clinically indicated.

### Death audit

All patients who, according to the prospective surveillance data, had had pure *P. vivax* infection at any stage during their final admission and who subsequently died at RSMM hospital between January 2004 and September 2009 were eligible for inclusion in the death audit. The data reviewed included 2,937 *P. vivax* infections and 46 deaths occurring between January 2004 and December 2007 that were included in a previous hospital-based epidemiological analysis [10]. Those diagnosed by RDT alone and those who only received a microscopic diagnosis of vivax malaria before referral to the hospital were excluded from the analysis. Data from the clinical notes were extracted by two physicians experienced in internal medicine. Information was recorded on a standardised death audit form and then entered into an EpiData database (version 3.1, EpiData Association, Odense, Denmark). The audit form documented presenting symptoms, past medical history, examination findings, laboratory and radiological investigation results, fulfilment of ‘severity’ criteria, diagnoses given during admission, treatment received and progress. If *Plasmodium* species identification was available from both hospital and research cross-check microscopy, the latter was taken as the best available evidence. For the purpose of study exclusion, a positive HRP2-based rapid antigen test (Paracheck Pf®, Orchid Biochemical Systems) was taken as confirmation of *P. falciparum* infection even if this was not detected on standard microscopy. If available, X-rays were interpreted by a certified infectious diseases specialist who was blinded to case history. Indicators of severity were only deemed to be present if the relevant criteria were fulfilled prior to the preterminal period (defined as within six hours of death).

After all relevant information had been extracted, three certified infectious diseases specialists (coauthors TWY, NMA and RNP) who had not been involved in the data extraction process independently reviewed the clinical data available for each case and subsequently classified the degree to which *P. vivax* was responsible for the deaths. This was done according to strict *a priori* criteria based on plausible pathological mechanisms reported in the scientific literature [16] and prior clinical experience with the disease (Table 1). Any disagreements in classification of the cause(s) of death were resolved by consensus. The sole exception to the criteria was for patients who did not fulfil the rules for inclusion in Category 1 (vivax malaria as primary cause of death) but who had no plausible alternative cause of death. In these instances, vivax malaria was classified as the prime cause of death. Although well-established to occur as a result of *P. vivax* infection alone [16], respiratory distress was only considered to be due to vivax malaria if there was no evidence of concomitant bacterial sepsis, conservatively defined as infiltrates on chest X-ray or any deviation in white cell count from the age-adjusted normal range [17]. Diarrhoea was conservatively attributed to alternative causes. Longitudinal cohorts indicate that *P. vivax* infection in children results in malnutrition [18], hence severe malnutrition with two or more documented episodes of vivax malaria in the preceding 12 months, was classified as vivax malaria being a major contributor to death.

**Table 1:** ***A priori*** **criteria for classifying cause of death**

**Category**	**Category description**	**Clinical criteria**
1	Pure vivax malaria the primary cause of death	Potential mechanisms include: coma, extreme anaemia (haemoglobin <3 g/dL), respiratory distress not associated with evidence of sepsis^a^, acidosis if associated with severe anaemia or splenic rupture
2	Pure vivax malaria likely to have been a major contributor to death	Alternative cause(s) more likely to have led to death but vivax malaria a major contributor through one of the following mechanisms: haemoglobin <7 g/dL, respiratory distress not associated with evidence of bacterial sepsis^a^, acidosis if associated with haemoglobin <7 g/dL, splenic rupture, decreased consciousness or malnutrition^b^ with two or more documented episodes of vivax malaria in the last year
3	Pure vivax malaria likely to have been a minor contributor to death	Alternative cause(s) more likely to have led to death but vivax malaria a minor contributor through one of the following mechanisms: fever, tachycardia or anaemia (haemoglobin between 7 g/dL and the lower limit of normal)
4	Pure vivax malaria unlikely to have contributed to death	No clear direct pathophysiological mechanism by which vivax malaria could have exacerbated or contributed to the primary cause(s) of death

^a^Sepsis conservatively attributed to bacterial co-infection. Evidence of sepsis defined as consolidation on chest X-ray or any deviation in white cell count from the age-adjusted normal range (birth; 20,000-40,000/mm^3^, 1 week; 5,000-21,000/mm^3^, 2 weeks; 5,000-20,000/mm^3^, 3 months to 12 months; 5,000-15,000/mm^3^, 1 year to 5 years; 5,000-12,000/mm^3^, greater than 5 years; 4,000-10,000/mm^3^).

^b^Malnutrition defined as documented malnutrition in the notes or a weight-for-age Z-score less than -3, according to the WHO Child Growth Standards [19].

### Statistical analyses

Data were analysed in STATA® version 10.1 (StataCorp, College Station, TX, USA) and EpiInfo® version 3.4.3 (Centers for Disease Control and Prevention, Atlanta, GA, USA). Proportions were compared using the chi-square test with Yates’ continuity correction or Fisher’s exact probability test and nonparametric continuous data, such as patient age, were compared using the Mann–Whitney U test. For the purposes of incidence and mortality rate estimation, we assumed a constant study population of 150,000 people (mid-way between the Indonesian government census estimates obtained in 2003 and 2007).

### Ethical considerations

This study was approved by the ethics committees of the University of Gadjah Mada, Indonesia (KE.FK.544.EC) and the Menzies School of Health Research, Australia (HREC 2010–1397). Since patients underwent no additional interventions above routine medical care, individual consent was not sought, unless the patient was enrolled in associated studies.

## Results

### The epidemiology of vivax malaria in Mimika District

**Figure 1:**
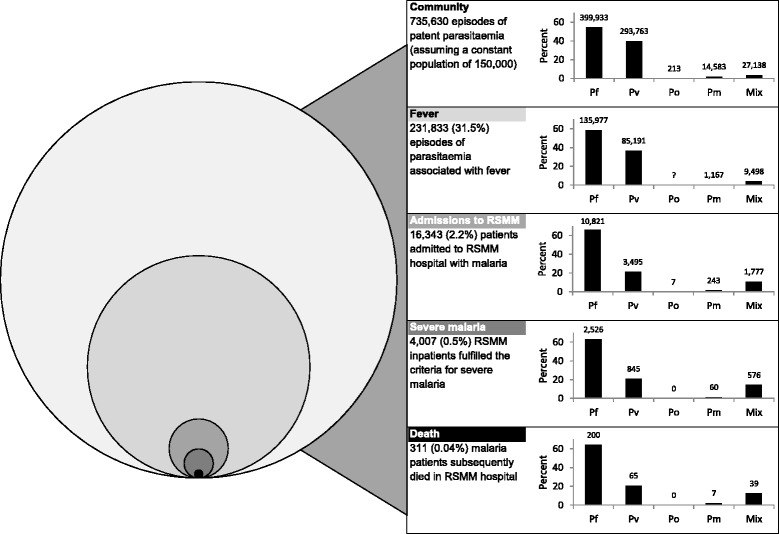
**Distribution of malaria cases, hospital admissions and deaths in Mimika District between January 2004 and September 2009 (to scale).** The figures for the community case work load (overall and febrile) were generated from the hospital surveillance as well as a previous prevalence and treatment seeking survey [13]. Abbreviations: Pf; *Plasmodium falciparum*, Pv; *Plasmodium vivax*, Po; *Plasmodium ovale*, Pm; *Plasmodium malariae*, Mix; mixed *Plasmodium* species infection.

**Figure 2:**
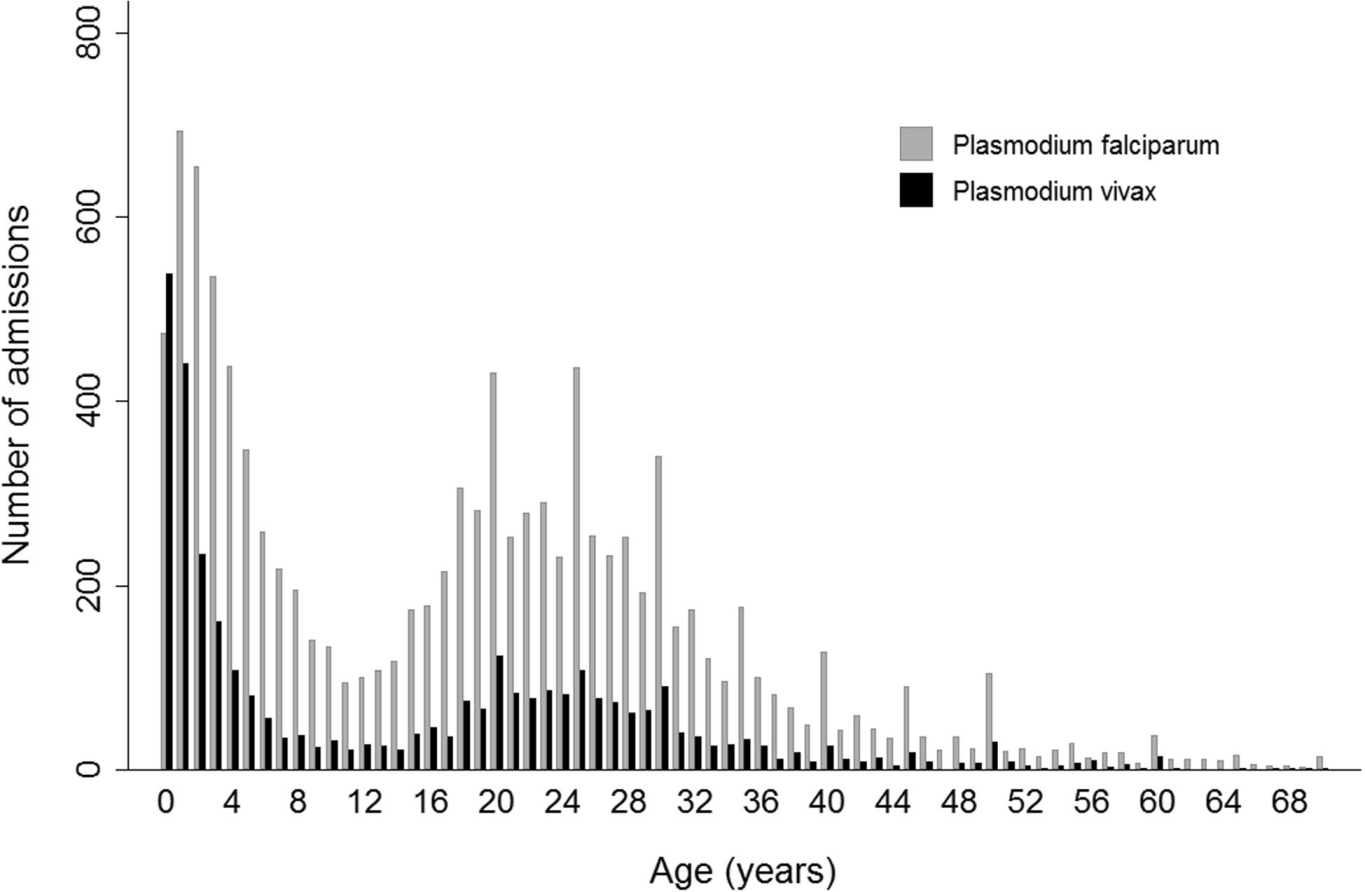
**Age distribution of patients admitted to Mitra Masyarakat Hospital with malaria between January 2004 and September 2009 by** ***Plasmodium*** **species.**

Between January 2004 and September 2009 there were an estimated 293,763 clinical or subclinical episodes of vivax malaria in Mimika District [13] resulting in 3,495 admissions to hospital with confirmed *P. vivax* parasitaemia (Figure 1). The number of patients admitted with malaria showed a bimodal age distribution with peaks between 0 to 10 and 15 to 35 years (Figure 2). The hospital surveillance system identified 845 (24%) patients admitted with *P. vivax* infection presenting with severe manifestations of malaria: anaemia in 19% (n = 652), respiratory distress in 5.3% (185) and coma in 1.6% (55). The corresponding figures for the 10,821 patients with *P. falciparum* infection were 23% (2,526) with severe manifestations: severe anaemia in 13% (1,451; *P* <0.001 for difference compared to *P. vivax*), respiratory distress in 5.0% (539, *P* = 0.5) and coma in 2.8% (298, *P* <0.001). During the same period there were 311 deaths in hospitalised patients with malaria, 65 (21%) of whom were reported to have had pure *P. vivax* parasitaemia. Based on these data, there were no differences in the age distribution (*P* = 0.74) or the proportion of females (*P* = 0.48) or non-Papuans (*P* = 0.90) in those who died with *P. vivax* parasitaemia versus those who survived.

### The death audit

**Figure 3:**
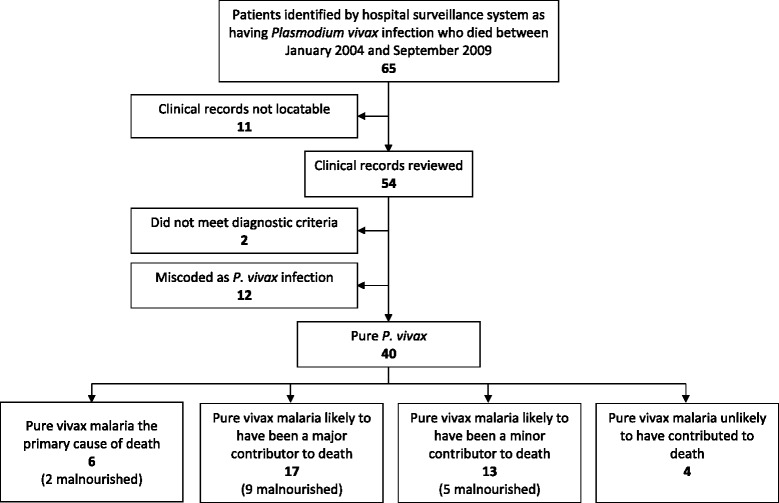
**Death audit profile.**

In total, 54 (83%) of the 65 relevant clinical charts were available for the death audit (Figure 3). The 11 patients for whom charts were not available had similar baseline characteristics to the remaining 54 patients. Two patients were excluded from the audit because they received their parasitological diagnoses outside of the hospital or their diagnoses were based exclusively on results from a RDT.

Blood films were available for cross-checking in 19% (10/52) of the remaining cases (Table 2) and altered the hospital diagnosis from *P. vivax* monoinfection to mixed *P. vivax/P. falciparum* infection in one case. Paracheck Pf® (Orchid Biochemical Systems) tests were performed in 19% (10/52) of cases and altered the diagnosis for one further case from *P. vivax* to mixed infection. On review of the notes and the above laboratory findings, a total of 12 of 52 cases (23%) had been incorrectly coded as infection with pure *P. vivax* infections. Six of these cases had been reclassified as mixed infections and six were due to coding errors. The remaining 40 (77%) patients had pure *P. vivax* parasitaemia according to the best available evidence. Chest X-rays were available for review in 26 of these cases.

**Table 2:** **Clinical details of the patients whose deaths were associated with vivax malaria**

**Category**	**No.**	**Age**	**Time to death**	**Weight (kg)**	**Documented malnutrition**	**Documented Pv in last year**	**Vivax parasitaemia**	**Haemoglobin (g/dL)**	**White cell count (μl** ^**-1**^ **)**	**Platelets × 10** ^**3**^ **μl** ^**-1**^	**Bilirubin (mmol/L)**	**Creatinine (μmol/L)**	**Glucose (mmol/L)**	**Bicarbonate (mmol/L)**	**CXR**	**Individual assessors’ classifications**	**Primary cause of death**	**Indirect causes**
**1. Primary cause**	1	19y	1d	40	N	2	++++	2.2	7,000	11	135.6	105	8.2	-	-	1,1,1	Vivax malaria with extreme anaemia associated with hyperbilirubinaemia	
	2^a^	1y7m	1d	7	Y	1	++	4.7	8,000	142	-	-	-	-	-	1,1,1	Vivax malaria	Malnutrition
	3	24y	4d	56	N	0	++++	2.6	16100	51	29.2	248	6.1	-	Resolving minor RLL consolidation compared with film 1 month prior	1,1,1	Vivax malaria with extreme anaemia and renal failure	
	4^b^	10 m	3d	7	Y	0	++++	1.9	11,800	64	-	-	13.5	3.0	Day 0 – normal Day 2 – whiteout of R lung and part of L lung. Pulmonary oedema in remainder of L lung	1,1,1	Vivax malaria with extreme anaemia and respiratory distress associated with acidosis	Malnutrition
	5	1y9m	2d	11	N	0	++++	1.7	20,100	139	116.3	-	5.6	-	-	1,1,1	Vivax malaria with extreme anaemia associated with hyperbilirubinaemia	Possible underlying sepsis
**2. Major contributor**	6	3m26d	5 h	-	N	0	+	2.8	29,000	123	-	-	6.5	-	Normal	1,1,2	Vivax malaria with extreme anaemia	Possible underlying sepsis
	7^a^	4y4m	2d	10	Y	0	++++	8.6	14,300	13	13.5	-	4.8	-	Normal	2,2,2	Respiratory tract infection	Malnutrition Vivax malaria
	8^a^	3y1m	3 h	12	N	0	++	-	-	-	-	-	-	-	Normal	2,2,2	Respiratory tract infection	Vivax malaria
	9	1y9m	7d	8	Y	3	+++	9.2	11,000	163	-	-	-	-	-	1,2,2	Malnutrition with sepsis	Vivax malaria
	10	1y8m	4d	8	Y	0	++++	5.1	4,200	154	-	-	-	-	Poor inspiratory effort, unable to assess	2,2,2	Respiratory tract infection	Malnutrition Vivax malaria
	11	1y8m	1d	-	Y	0	+++	7.3	13,900	13	-	56	21.8		Normal	1,2,3	Respiratory tract infection	Vivax malaria Malnutrition
	12^a^	1y3m	9d	6	Y	3	+++	12.5	25,300	136	5.3	-	6.8		-	2,2,3	Diarrhoeal disease	Malnutrition Vivax malaria
	13	1y	7d	6	Y	0	++	7.9	8,300	105	-	43	4.6	9.8	Normal	3,2,2	Diarrhoeal disease associated with acidosis	Vivax malaria Malnutrition
	14	38y	13d	26	Y	0	+++	6.3	14,300	55	-	106	6.0	-	Minor chronic changes in LLL	2,2,2	AIDS	Vivax malaria Malnutrition
	15	2y	2d	12	Y	0	+	4.4	10,600	21	23.4	48	4.3	26.7	Evidence of pneumonia	2,2,2	Respiratory tract infection	Vivax malaria Malnutrition
	16	1y5m	17d	5	Y	3	++	6.7	9,500	262	-	-	6.0	-	Diffuse changes consistent with pneumonia, R > L	2,2,2	AIDS	Vivax malaria Malnutrition Tuberculosis
	17	27y	5d	-	N	1	+	6.5	3,800	80	-	-	-	19.2	-	2,2,2	AIDS	Vivax malaria
	18	1y23d	6 h	9	N	0	+++	5.8	4,700	229	-	44	8.7	9.3	RUL consolidation	2,2,2	Bronchopneumonia associated with acidosis	Vivax malaria
	19	34y	8 h	50	N	0	++	5.8	14,100	252	-	-	4.9	2.2	Bilaterally increased lung markings, R > L, consistent with ARDS or pneumonia	2,2,2	Chronic renal failure associated with acidosis	Vivax malaria Respiratory tract infection
	20^b^	61y	6d	50	N	0	+	4.1	3,500	9	21.7	2378	5.3	13.6	RML collapse with consolidation, calcification and scarring	2,2,2	Chronic renal failure with respiratory tract infection associated with acidosis	Vivax malaria
	21^a,b^	25y	5 h	75	N	0	+	6.2	23,700	22	-	97	15.4	-	-	2,2,2	GI bleeding secondary to NSAID	Vivax malaria
	22^b^	3y	1d	11	N	0	++	9.4	9,600	152	-	114	1.7	13.3	Admission – underexposed but probably normal Later in the day – collapsed L lung with ETT in R main bronchus. R lung normal	2,2,2	Sepsis associated with hypoglycaemia and acidosis	Vivax malaria Trauma
	23^b^	14y	8d	-	N	0	+++	2.1	4,200	89	19.0	1680	-	2.2	-	2,2,3	Uraemia secondary to endstage chronic renal failure associated with acidosis	Vivax malaria exacerbating severe anaemia
**3. Minor contributor**	24	2y4m	6d	9	Y	1	++	13.0	12,600	231	40.4	-	6.9	-	-	2,3,3	Diarrhoeal disease	Sepsis Malnutrition Vivax malaria
	25	59y	3d	50	N	0	+	10.5	35,500	173	33.5	228	4.2	-	-	3,3,3	Respiratory sepsis	COPD Vivax malaria Ischemic heart disease
	26	2y10m	5d	12	Y	1	+	9.0	13200	124	-	12	5.8	22.8	Dense, bilateral patchy consolidation L > R	3,3,3	Tuberculosis with sepsis	Vivax malaria Malnutrition
	27^a^	18y	1d	50	N	0	++	15.5	16,100	100	-	332	11.4	17.1	Normal	3,3,3	Meningitis associated with renal failure	Vivax malaria
	28^a^	60y	1d	37	N	0	+	11.5	11,800	119	-	600	5.7	-	-	3,3,4	Hepatorenal syndrome	Vivax malaria Possible bacterial infection
	29	1y8m	1d	7	N	1	++	9.8	79,300	580	-	-	2.4	9.9	CXR 2 days prior showed infiltrate obscuring L heart border	3,3,3	Respiratory tract infection with overwhelming sepsis and acidosis	Vivax malaria Malnutrition
	30	24y	5d	33	N	2	+	4.7	1,500	85	6.7	132	5.7	21.9	Worsening RML consolidation and cavitation compared with previous films. L mid and lower zone consolidation and cavitation. R mediastinal mass	3,3,3	AIDS with tuberculosis	Vivax malaria
	31	5m4d	12d	7	N	0	++	13.0	7,200	107	-	-	-	13.9	Infiltrate at R hilum	3,4,3	Bronchopneumonia associated with acidosis	Vivax malaria
	32	1y8m	1d	6	Y	0	++++	10.2	19,700	110	-	66	1.2	-	Normal	3,3,3	Diarrhoeal disease with hypovolaemic shock	Vivax malaria Malnutrition
	33	55y	4d	60	N	0	++++	10.1	19,500	55	-	521	3.7	-	Increased lung markings bilaterally	3,3,3	Sepsis and renal failure	Vivax malaria
	34	4y	1d	16	N	0	+	11.2	22,700	461	-	-	6.0	-	-	3,3,3	GI sepsis	Vivax malaria
	35	31y	10d	38	Y	1	+	9.9	3,700	114	3.4	131	4.6	-	-	3,3,3	HIV associated enteropathy	Malnutrition Vivax malaria
	36	3y6m	15d	12	Y	2	++	8.9	27,700	3	63.3	-	-	33.2	Poor inspiratory effort, unable to assess	3,3,4	Respiratory tract infection with overwhelming sepsis associated with hyperbilirubinaemia	Malnutrition Vivax malaria

^a^Research microscopy done; ^b^Paracheck Pf® done. AIDS; acquired immunodeficiency syndrome, ARDS; acute respiratory distress syndrome, CXR; chest X-ray, d; day, h; hour, ETT; endotracheal tube, GI; gastrointestinal, HIV; human immunodeficiency virus, L; left, LLL; left lower lobe, m; month, NSAID; non-steroidal anti-inflammatory drug, Pv; *Plasmodium vivax*, R; right, RLL; right lower lobe, RML; right middle lobe, RUL; right upper lobe, y; year. To comply with the BMC policy on patient confidentiality, details on gender were removed from the table to ensure there were less than three indirect identifiers per patient.

Four patients died of causes thought unlikely to be related to *P. vivax* infection (Figure 3); two due to road traffic accidents, one due to electrocution and one due to tuberculous meningoencephalitis. These patients were excluded from further analysis. The remaining patients were classified as Category 1 (*P. vivax* infection the prime cause of death) in 6 cases, Category 2 (*P. vivax* infection a major contributor) in 17 cases and Category 3 (*P. vivax* infection a minor contributor) in 13 cases. Of these 36 patients, 23 (64%) had presented to the hospital in the 12 months preceding their death. The median number of preceding presentations was 5 (range 1 to 17) and the proportion of these presentations due to malaria was 25% (41/161). Malnutrition was recorded in 16 (44%) patients and was present in 64% (14/22) of children less than five-years old who died compared to 14% (2/14) of those five-years old and older. Patients who had presented to the hospital with malaria over the preceding 12 months were at greater risk of malnutrition (59%, 10/17) compared to those with no documentation of prior presentation with malaria (31%, 6/19), although this did not reach statistical significance (*P* = 0.18).

The median age of the patients in Category 1 was 1.7 years (interquartile (IQ) range 0.71 to 21 years), 3.0 years (IQ range 1.5 to 26 years) in Category 2 and 4 years (IQ range 2.0 to 43 years) in Category 3, *P = *0.23. There was a predominance of females in all three categories (83% (5/6), 77% (13/17) and 54% (7/13), respectively), although this was also the case in hospitalised patients with vivax malaria as a whole (60% (2,081/3,495)).

Overall, the most prominent manifestation of severity was respiratory distress which was present in 74% (26) of the 35 patients in whom this could be assessed. In total, 83% (19/23) of children (≤15 years) had respiratory distress compared with 58% (7/12) of adults; *P* = 0.36. Respiratory distress was present in all of the ten patients with severe anaemia compared to only 62% (15/24) of those patients without severe anaemia; *P* = 0.07.

Four (13%) of the 31 patients in whom a Glasgow or Blantyre Coma Score was reported fulfilled criteria for cerebral malaria prior to the preterminal period (Table 2 patients #14, #22, #27 and #32). In one case a lumbar puncture was suggestive of bacterial meningitis. The other three deaths were not deemed to be primarily attributable to vivax malaria.

**Figure 4:**
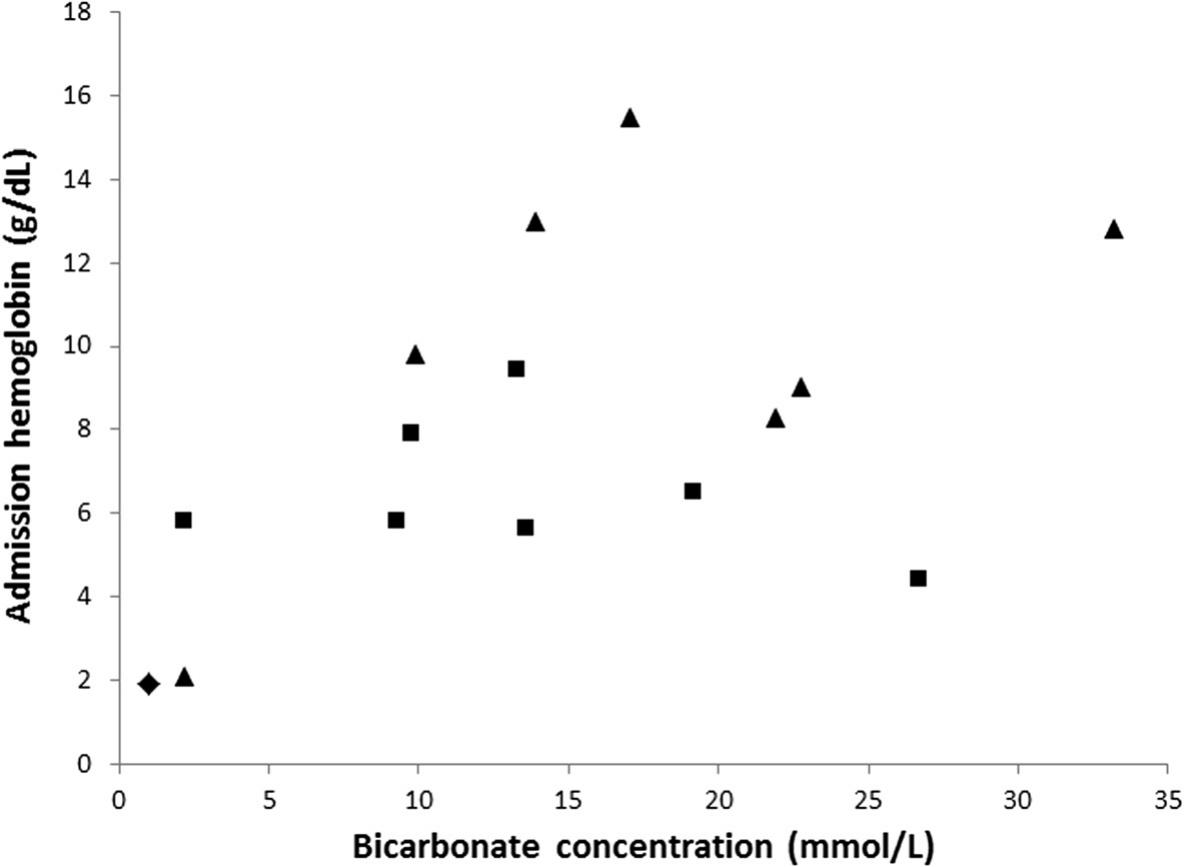
**Relationship between anaemia and acidosis in vivax-associated deaths.** Diamond = Category 1, Squares = Category 2 and Triangles = Category 3. r_s_ = 0.45, *P* = 0.09.

Haematology testing was available in 97% (35/36) of patients in the death audit. There was a non-statistically significant association between anaemia and acidosis (r_s_ = 0.45, *P* = 0.09; Figure 4). Severe anaemia was present in 29% (10/35) and acidosis in 64% (9/15) of patients who had blood gas analyses (three of the acidotic patients also had severe anaemia and eight had respiratory distress). Severe thrombocytopaenia was relatively common with 19% (7/35) of patients having a platelet concentration of less than 50 × 10^9^ per litre and 14% (5/35) less than 20 × 10^9^ per litre. Three of the 13 patients tested (23%) fulfilled the WHO criterion for hyperbilirubinaemia (>42.75 mmol/L), two of whom succumbed to vivax malaria as a direct cause of death. Two of the 28 patients assessed (7%) had hypoglycaemia (glucose <2.2 mmol/L). Six of nineteen patients assessed (32%) had a creatinine concentration above 230 μmol/L (the WHO criterion for severe malaria).

Three recurring patterns, and potential mechanisms, of death emerged. The first mechanism was extreme anaemia (haemoglobin <3 g/dL) which was deemed to be the cause of death in five of the six (83%) patients in Category 1. The second was a combination of severe malnutrition and vivax malaria, often with additional sepsis with respiratory or gastrointestinal manifestations. Overall, 16 (44%) of the 36 vivax-associated deaths were in patients with documented malnutrition, including three patients who had a WHO weight-for-age Z-score of less than −3 [19]. The third pattern constituted vivax malaria in association with chronic or subacute morbidity, such as chronic kidney disease (n =3), HIV infection (n =5) or active tuberculosis (n =2).

### Overall risks

After correcting for errors in parasite species identification, *P. vivax* was found to have caused or contributed to a minimum of 36 deaths over the 69-month accrual period, corresponding to a minimum case fatality of 10.3 per 1,000 (36/3,495) hospitalised patients with *P. vivax* infection (Table 3). From the concurrent community surveillance and a household survey of treatment seeking behaviour [13] an estimated 293,763 cases of vivax parasitaemia occurred in Mimika District over the study accrual period, giving rise to an overall estimated minimum case fatality rate of 0.12 per 1,000 infections. The upper limits for the hospital and community fatality estimates were 16.9 and 0.63 per 1,000, respectively (see the footnote of Table 3 for the assumptions underlying this sensitivity analysis). The case fatality of vivax malaria in children under five years of age compared with those five years of age or older was 14.9 versus 7.0 per 1,000 in the hospital and 0.26 versus 0.07 per 1,000 in the community.

**Table 3:** **Case fatality per 1,000 patients with vivax malaria**

	**Hospitalised patients**		**Overall population** ^**b**^	
**Age group**	**Minimum (n/D)**	**Upper limit** ^**a**^ **(n/D)**	**Minimum (n/D)**	**Upper limit (n/D)**
<5 years	14.9	19.6	0.26	1.13
	(95% CI 9.3 to 22.4)	(95% CI 13.1 to 28.0)	(95% CI 0.16 to 0.40)	(95% CI 0.87 to 1.33)
	(22/1,480^c^)	(29/1,480)	(22/84,028)	(90.6/84,028)
≥5 years	7.0	15.1	0.07	0.45
	(95% CI 3.9 to 11.8)	(95% CI 10.2 to 21.5)	(95% CI 0.04 to 0.11)	(95% CI 0.36 to 0.55)
	(14/1,986^c^)	(30/1,986)	(14/209,735)	(93.8/209,735)
All	10.3	16.9	0.12	0.63
	(95% CI 7.2 to 14.20	(95% CI 12.9 to 21.7)	(95% CI 0.09 to 0.17)	(95% CI 0.54 to 0.72)
	(36/3,495)	(59/3,495)	(36/293,763)	(184.4/293,763)

^a^Upper limits were calculated assuming the following: 1) all of the patients for whom notes were not available died of causes related to *P. vivax* infection; 2) an equal number of deaths were miscoded as being attributable to *P. falciparum* as were miscoded as being attributable to *P. vivax*; ^b^denominators include hospital and community patients with *P. vivax*, estimated from the total number of cases seen in our community surveillance network multiplied by the reciprocal of the proportion who sought treatment at our network facilities (40%, established from a house-to-house survey of treatment seeking behaviour [3]). To calculate the upper limits, the same assumptions were made as in the hospitalised patients except we also multiplied the number of deaths by the reciprocal of 32% (the proportion of deaths estimated to occur outside of hospital (unpublished data)); ^c^a small proportion of individuals in the active surveillance had an unknown age. N; number, D; denominator.

## Discussion

Over recent years, malariometric surveillance in southern Papua has identified a significant number of individuals who have died following *P. vivax* infection. Our previous publication based on an earlier set of surveillance data reported an overall risk of death in patients admitted with vivax malaria of 1.6%, raising significant concerns that the virulence of this organism had been underestimated [10]. In the current analysis we used *a priori* definitions to classify the contribution of vivax malaria to those deaths. We excluded individuals with mixed *Plasmodium* species infections and used conservative criteria to define potential concomitant bacterial sepsis. Our results highlight that over a six year period, a minimum of 36 deaths could be at least partially attributed to *P. vivax* infection and that in six cases vivax malaria was most likely the primary cause of death. This resulted in a fatality of 1 in 97 for patients admitted to hospital and 1 in 8,160 for the community as a whole. Children younger than five years old were at a two- to four-fold greater risk of death compared to those older than five years.

Five of the patients in the study died of extreme anaemia (probably as a result of high-output cardiac failure and acidosis), while severe anaemia was a major contributor in five other fatal cases. A study from Venezuela suggests that in areas of mixed species endemicity, anaemia may be a more frequent and severe sequel of vivax compared with falciparum malaria [20]. In Timika, the mean reduction in haemoglobin and risk of severe anaemia is greater for infants (<1 year of age) infected with *P. vivax* when compared with *P. falciparum* and, thereafter, the haematologic impact of *P. falciparum* exceeds that of *P. vivax,* [3,21]. We have shown previously that 12% of all deaths at the RSMM are attributable to patients with severe anaemia and overall 6% of all cases of severe anaemia are attributed to vivax malaria [3].

Overall, nearly half (44%) of the vivax-associated deaths had evidence of malnutrition and a high proportion of these individuals also had clinical or radiological evidence of possible concomitant bacterial infection. Chronic *P. vivax* infections have been implicated in a protein-wasting condition akin to kwashiorkor, in both induced [22] and natural infection [23]. Indeed, the evidence that *P. vivax* causes malnutrition is stronger than that for *P. falciparum* [24]. It is also likely that there is an epidemiological, and possibly a biological, association between malnutrition and a greater risk of *P. vivax* severe disease [25], as has been noted previously in patients infected with *P. falciparum* [26]. While this relationship is likely bidirectional, our study supports previous literature suggesting that malnutrition significantly worsens the outcome of infectious disease and implicates vivax malaria as not only a potential cause of malnutrition, but also a subsequent precipitating cause of death in those with significant malnutrition [16].

More than 70% of audited individuals fulfilled the criteria for respiratory distress. Previous work has suggested that vivax-associated lung dysfunction may occur from an inflammatory response in the pulmonary microvasculature, exacerbated by treatment [27], causing increased capillary permeability, alveolar endothelial damage and chest X-ray infiltrates [28-30]. One infant had an acute respiratory distress-type picture. Bilateral lung infiltrates consistent with acute lung injury were seen in 15% of all adult vivax-associated deaths and in a third of those who had chest X-rays. Severe anaemia and metabolic acidosis are also likely to be key determinants, particularly in children, in whom lung injury is less common than in adults. Eight of the nine patients with acidosis were found to be in respiratory distress as were all ten patients with severe anaemia.

Studies of *P. falciparum* infection in African children have shown high coprevalence of pneumonia, meningitis and bacteraemia in children with severe malaria [31-33], the largest of which demonstrated a strong association between coinfection and an increased risk of death [33]. A study in Papua New Guinea has also highlighted the importance of respiratory distress in vivax malaria, although the degree to which this was attributable to *P. vivax* infection, associated anaemia or comorbid pneumonia was unclear [34]. Since microbiological data were not available in our study, we conservatively regarded any deviation in white cell count or infiltrates on chest X-ray as evidence of concomitant bacterial infection. In other studies, albeit without routine blood cultures for bacteraemia, severe and fatal sepsis syndromes have been attributed to vivax malaria [35]. Given the potential for *P. vivax* to cause an intense inflammatory response [36] and/or lung injury/infiltrates [27] it is likely that we have significantly overestimated the contribution of infectious comorbidities and underestimated the direct contribution of vivax malaria alone to sepsis, lung injury and death. Nevertheless, our results suggest that, as with falciparum malaria, there may be a potentially fatal interaction between vivax malaria and bacterial infection, particularly pneumonia. To clarify this interaction, prospective studies of severe vivax malaria syndromes need to include systematic investigation for concomitant bacterial infection, including blood cultures [37]. Until then, our findings suggest the need for aggressive antimalarial treatment in patients with evidence of coinfection, and also indicate that mono-infected patients with vivax malaria who show signs of severe disease should be investigated and treated for associated bacterial sepsis.

HIV infection is now recognised as a major contributor to the development of severe and fatal disease in *P. falciparum* malaria [38]. Fourteen percent of vivax-associated deaths were in patients with documented HIV co-infection, which, with the recent rapid increase in HIV infection in Papua [39] and the retrospective study design, is likely to be an underestimate of its contribution. While *P. vivax* may act as the final precipitating cause of death in HIV infected individuals, a potential role for HIV in exacerbating the severity of vivax malaria, as occurs with *P. falciparum*, requires further investigation.

Our study has several important limitations. According to laboratory reports, 12 of the 54 patients in the death audit were actually shown to be suffering from *P. falciparum* or mixed species infections. Since we did not systematically review all *P. falciparum* deaths, it is possible that similar miscoding occurred in the opposite direction (for example low density *P. vivax* parasitaemia missed on blood smear examination). This would have resulted in an underestimation of the true incidence of deaths due to *P. vivax* infection. Our sensitivity analysis reported in Table 3 assumes that an equal number of deaths were miscoded as being attributable to *P. falciparum* as were miscoded as being attributable to *P. vivax*.

Differentiating *P. vivax* from *P. falciparum* early ring (trophozoite) stages using standard microscopy is fraught with error. Despite high concordance (90%) of hospital microscopy results and an independent expert’s findings, it is likely that a degree of misdiagnosis occurred, even in cases where cross-check microscopy was performed. Fully quantifiable parasitaemias were not available to the adjudicating physicians; however, the semi-quantitative scoring gave an indication of relative parasite density.

Rapid diagnostic tests were only done in a minority of cases. Most HRP2-based tests have been shown to be highly sensitive for the detection of falciparum malaria; therefore, a significant biomass of *P. falciparum* would have been unlikely in patients who tested negative [40]. For the remaining patients, it is possible that microscopy may have missed subpatent *P. falciparum* parasitaemias. Siripoon *et al.* found that 13% of patients who had been diagnosed with *P. vivax* monoinfections in Thailand also had subpatent *P. falciparum* infections based on PCR testing [41]. Because PCR diagnostics are still not widely available in endemic areas, this is unlikely to have a bearing on the clinical classification of malaria or its treatment. It does, however, limit our ability to categorically incriminate *P. vivax* monoinfection as a cause of fatal disease.

Previous treatment seeking studies in southern Papua estimate that almost 68% of deaths occur outside of hospital (unpublished data). We incorporated this crude proportion in the sensitivity analysis but were unable to make more subtle adjustments based on differences in the demographic and causative distribution of those deaths.

## Conclusion

We have shown that in southern Papua, mortality primarily attributable to *P. vivax* infection may occur but that indirect contribution to death in those with comorbidities, such as malnutrition, HIV and possible bacterial sepsis, is a more common scenario. Future research should include postmortem studies and detailed clinical and microbiological investigations of severe cases of vivax malaria to characterise pathogenic mechanisms and elucidate better therapeutic strategies by which fatal outcomes may be averted [42]. If the overall burden of *P. vivax* is to be appreciably decreased, practical, short-course, sterilising drug regimens must be developed that reliably prevent relapses and reduce transmission of this complicated disease.

## References

[1] Guerra CA, Howes RE, Patil AP, Gething PW, Van Boeckel TP, Temperley WH, Kabaria CW, Tatem AJ, Manh BH, Elyazar IR, Baird JK, Snow RW, Hay SI (2010). The international limits and population at risk of Plasmodium vivax transmission in 2009. PLoS Negl Trop Dis.

[2] Gething PW, Elyazar IR, Moyes CL, Smith DL, Battle KE, Guerra CA, Patil AP, Tatem AJ, Howes RE, Myers MF, George DB, Horby P, Wertheim HF, Price RN, Müeller I, Baird JK, Hay SI (2012). A long neglected world malaria map: Plasmodium vivax endemicity in 2010. PLoS Negl Trop Dis.

[3] Douglas NM, Lampah DA, Kenangalem E, Simpson JA, Poespoprodjo JR, Sugiarto P, Anstey NM, Price RN (2013). Major burden of severe anemia from non-falciparum malaria species in Southern Papua: a hospital-based surveillance study. PLoS Med.

[4] Poespoprodjo JR, Fobia W, Kenangalem E, Lampah DA, Warikar N, Seal A, McGready R, Sugiarto P, Tjitra E, Anstey NM, Price RN (2008). Adverse pregnancy outcomes in an area where multidrug-resistant plasmodium vivax and Plasmodium falciparum infections are endemic. Clin Infect Dis.

[5] McGready R, Lee SJ, Wiladphaingern J, Ashley EA, Rijken MJ, Boel M, Simpson JA, Paw MK, Pimanpanarak M, Mu O, Singhasivanon P, White NJ, Nosten FH (2012). Adverse effects of falciparum and vivax malaria and the safety of antimalarial treatment in early pregnancy: a population-based study. Lancet Infect Dis.

[6] Baird JK (2013). Evidence and implications of mortality associated with acute Plasmodium vivax malaria. Clin Microbiol Rev.

[7] Price RN, Douglas NM, Anstey NM (2009). New developments in Plasmodium vivax malaria: severe disease and the rise of chloroquine resistance. Curr Opin Infect Dis.

[8] Barcus MJ, Basri H, Picarima H, Manyakori C (2007). Sekartuti, Elyazar I, Bangs MJ, Maguire JD, Baird JK: **Demographic risk factors for severe and fatal vivax and falciparum malaria among hospital admissions in northeastern Indonesian Papua**. Am J Trop Med Hyg.

[9] Rodriguez-Morales AJ, Benitez JA, Arria M (2008). Malaria mortality in Venezuela: focus on deaths due to plasmodium vivax in children. J Trop Pediatr.

[10] Tjitra E, Anstey NM, Sugiarto P, Warikar N, Kenangalem E, Karyana M, Lampah DA, Price RN (2008). Multidrug-resistant plasmodium vivax associated with severe and fatal malaria: a prospective study in Papua. Indonesia. PLoS Med.

[11] Lacerda MV, Fragoso SC, Alecrim MG, Alexandre MA, Magalhaes BM, Siqueira AM, Ferreira LC, Araujo JR, Mourao MP, Ferrer M, Castillo P, Martin-Jaular L, Fernandez-Becerra C, del Portillo H, Ordi J, Alonso PL, Bassat Q (2012). Postmortem characterization of patients with clinical diagnosis of Plasmodium vivax malaria: to what extent does this parasite kill?. Clin Infect Dis.

[12] Ratcliff A, Siswantoro H, Kenangalem E, Maristela R, Wuwung RM, Laihad F, Ebsworth EP, Anstey NM, Tjitra E, Price RN (2007). Two fixed-dose artemisinin combinations for drug-resistant falciparum and vivax malaria in Papua, Indonesia: an open-label randomised comparison. Lancet.

[13] Karyana M, Burdarm L, Yeung S, Kenangalem E, Wariker N, Maristela R, Umana KG, Vemuri R, Okoseray MJ, Penttinen PM, Ebsworth P, Sugiarto P, Anstey NM, Tjitra E, Price RN (2008). Malaria morbidity in Papua Indonesia, an area with multidrug resistant Plasmodium vivax and Plasmodium falciparum. Malar J.

[14] Randall LM, Kenangalem E, Lampah DA, Tjitra E, Mwaikambo ED, Handojo T, Piera KA, Zhao ZZ, de Labastida Rivera F, Zhou Y, McSweeney KM, Le L, Amante FH, Haque A, Stanley AC, Woodberry T, Salwati E, Granger DL, Hobbs MR, Price RN, Weinberg JB, Montgomery GW, Anstey NM, Engwerda CR (2010). Age-related susceptibility to severe malaria associated with galectin-2 in highland Papuans. J Infect Dis.

[15] **Severe falciparum malaria. World Health Organization, Communicable Diseases Cluster.** *Trans R Soc Trop Med Hyg* 2000, **94:**S1–S90. 11103309

[16] Anstey NM, Douglas NM, Poespoprodjo JR, Price RN (2012). Plasmodium vivax: clinical spectrum, risk factors and pathogenesis. Adv Parasitol.

[17] Dipchand AI (1997). The HSC Handbook of Pediatrics.

[18] Lee G, Yori P, Olortegui MP, Pan W, Caulfield L, Gilman RH, Sanders JW, Delgado HS, Kosek M (2012). Comparative effects of vivax malaria, fever and diarrhoea on child growth. Int J Epidemiol.

[19] **The WHO Child Growth Standards.** [http://www.who.int/childgrowth/en/]

[20] Rodriguez-Morales AJ, Sanchez E, Vargas M, Piccolo C, Colina R, Arria M, Franco-Paredes C (2006). Is anemia in Plasmodium vivax malaria more frequent and severe than in Plasmodium falciparum?. Am J Med.

[21] Poespoprodjo JR, Fobia W, Kenangalem E, Lampah DA, Hasanuddin A, Warikar N, Sugiarto P, Tjitra E, Anstey NM, Price RN (2009). Vivax malaria: a major cause of morbidity in early infancy. Clin Infect Dis.

[22] Kitchen SF (1939). Malariology.

[23] Hutchinson RA, Lindsay SW (2006). Malaria and deaths in the English marshes. Lancet.

[24] Williams TN, Maitland K, Phelps L, Bennett S, Peto TE, Viji J, Timothy R, Clegg JB, Weatherall DJ, Bowden DK (1997). Plasmodium vivax: a cause of malnutrition in young children. QJM.

[25] Lanca EF, Magalhaes BM, Vitor-Silva S, Siqueira AM, Benzecry SG, Alexandre MA, O’Brien C, Bassat Q, Lacerda MV (2012). Risk factors and characterization of Plasmodium vivax-associated admissions to pediatric intensive care units in the Brazilian Amazon. PLoS One.

[26] Berkley JA, Bejon P, Mwangi T, Gwer S, Maitland K, Williams TN, Mohammed S, Osier F, Kinyanjui S, Fegan G, Lowe BS, English M, Peshu N, Marsh K, Newton CR (2009). HIV infection, malnutrition, and invasive bacterial infection among children with severe malaria. Clin Infect Dis.

[27] Anstey NM, Handojo T, Pain MC, Kenangalem E, Tjitra E, Price RN, Maguire GP (2007). Lung injury in vivax malaria: pathophysiological evidence for pulmonary vascular sequestration and posttreatment alveolar-capillary inflammation. J Infect Dis.

[28] Tan LK, Yacoub S, Scott S, Bhagani S, Jacobs M (2008). Acute lung injury and other serious complications of Plasmodium vivax malaria. Lancet Infect Dis.

[29] Price L, Planche T, Rayner C, Krishna S (2007). Acute respiratory distress syndrome in Plasmodium vivax malaria: case report and review of the literature. Trans R Soc Trop Med Hyg.

[30] Taylor WR, Hanson J, Turner GD, White NJ, Dondorp AM (2012). Respiratory manifestations of malaria. Chest.

[31] Bronzan RN, Taylor TE, Mwenechanya J, Tembo M, Kayira K, Bwanaisa L, Njobvu A, Kondowe W, Chalira C, Walsh AL, Phiri A, Wilson LK, Molyneux ME, Graham SM (2007). Bacteremia in Malawian children with severe malaria: prevalence, etiology, HIV coinfection, and outcome. J Infect Dis.

[32] Berkley JA, Mwangi I, Mellington F, Mwarumba S, Marsh K (1999). Cerebral malaria versus bacterial meningitis in children with impaired consciousness. QJM.

[33] Gwer S, Newton CR, Berkley JA (2007). Over-diagnosis and co-morbidity of severe malaria in African children: a guide for clinicians. Am J Trop Med Hyg.

[34] Genton B, D’Acremont V, Rare L, Baea K, Reeder JC, Alpers MP, Muller I (2008). Plasmodium vivax and mixed infections are associated with severe malaria in children: a prospective cohort study from Papua New Guinea. PLoS Med.

[35] Andrade BB, Reis-Filho A, Souza-Neto SM, Clarencio J, Camargo LM, Barral A, Barral-Netto M (2010). Severe Plasmodium vivax malaria exhibits marked inflammatory imbalance. Malar J.

[36] Yeo TW, Lampah DA, Tjitra E, Piera K, Gitawati R, Kenangalem E, Price RN, Anstey NM (2010). Greater endothelial activation, Weibel-Palade body release and host inflammatory response to Plasmodium vivax, compared with Plasmodium falciparum: a prospective study in Papua, Indonesia. J Infect Dis.

[37] Bhattacharya SK, Sur D, Dutta S, Kanungo S, Ochiai RL, Kim DR, Anstey NM, von Seidlein L, Deen J (2013). Vivax malaria and bacteraemia: a prospective study in Kolkata. India Malar J.

[38] Bejon P, Berkley JA, Mwangi T, Ogada E, Mwangi I, Maitland K, Williams T, Scott JA, English M, Lowe BS, Peshu N, Newton CR, Marsh K (2007). Defining childhood severe falciparum malaria for intervention studies. PLoS Med.

[39] Pontororing GJ, Kenangalem E, Lolong DB, Waramori G, Sandjaja, Tjitra E, Price RN, Kelly PM, Anstey NM, Ralph AP, Sandjaja SS, Tjitra E, Price RN, Kelly PM, Anstey NM, Ralph AP (2010). The burden and treatment of HIV in tuberculosis patients in Papua Province, Indonesia: a prospective observational study. BMC Infect Dis.

[40] Swarthout TD, Counihan H, Senga RK, van den Broek I (2007). Paracheck-Pf accuracy and recently treated Plasmodium falciparum infections: is there a risk of over-diagnosis?. Malar J.

[41] Siripoon N, Snounou G, Yamogkul P, Na-Bangchang K, Thaithong S (2002). Cryptic Plasmodium falciparum parasites in clinical P. vivax blood samples from Thailand. Trans R Soc Trop Med Hyg.

[42] Barber BE, William T, Grigg MJ, Parameswaran U, Piera KA, Price RN, Yeo TW, Anstey NM: **Parasite Biomass-Related Inflammation, and Endothelial Activation, Microvascular Dysfunction and Disease Severity in Vivax Malaria.** *PLoS Pathogens,* in Press 2014. 10.1371/journal.ppat.1004558 PMC4287532 25569250

